# Feasibility of quick response-based quality improvement projects in an urban primary care setting: A cross-sectional survey

**DOI:** 10.51866/oa.653

**Published:** 2024-10-03

**Authors:** Zi-Yi Yeoh, Hooi Chin Beh, Megat Mohamad Amirul Amzar Megat Hashim, Abdul Hadi Haireen, Deik Roy Chuan, Sajaratulnisah Othman

**Affiliations:** 1 MBBC, MFamMed, PhD, Department of Primary Car Medicine, Faculty of Medicine, Universiti Malaya, Kuala Lumpur, Malaysia. Email: sajaratul@ummc.edu.my; 2 MBBS, MFamMed, Department of Primary Care Medicine, Faculty of Medicine, Universiti Malaya, Kuala Lumpur, Malaysia. Email: hcbeh@ummc.edu.my; 3 MB ChB, Department of Primary Care Medicine, Universiti Malaya Medical Centre, Kuala Lumpur, Malaysia.; 4 MBBS, MFamMed, Department of Primary Care Medicine, Universiti Malaya Medical Centre, Kuala Lumpur, Malaysia.; 5 MB BCh BAO, BMEDSC, MFamMed, Department of Primary Care Medicine, Faculty of Medicine, Universiti Malaya, Kuala Lumpur, Malaysia.; 6 MBBS, Department of Primary Care Medicine, Universiti Malaya Medical Centre, Kuala Lumpur, Malaysia.

**Keywords:** Digital technology, Educational technology, Health facility administration

## Abstract

**Introduction:**

Using quick response (QR) codes to disseminate information has become increasingly popular since the declaration of COVID-19 as a pandemic. We aimed to investigate the feasibility of implementing QR-based quality improvement projects in our clinic to improve patients’ medical knowledge, experience and access to care.

**Methods:**

We utilised systematic random sampling by recruiting every 25th patient registered in our clinic during data collection. Participants answered a self-administered printed questionnaire regarding their smartphone usage and familiarity with QR code scanning at the patients’ waiting area. Data were analysed using the Statistical Package for the Social Sciences version 26.

**Results:**

A total of 323 patients participated (response rate=100%). The participants’ median age was 57 years (interquartile range=4l-67). Most participants were women (63.1%). Approximately 90.4% (n=282) used smartphones, with 83.7% (n=261) reporting average or good usage proficiency. More than half (58.0%) accessed medical information via their smartphones, and 67.0% were familiar with QR codes. Multiple logistic regression analyses revealed that familiarity with QR codes was linked to age of <65 years [adjusted odds ratio (AOR)=4.593, 95% confidence interval (CI)=2.351-8.976, P<0.001], tertiary education (AOR=2.385, 95% CI=1.170—4.863, P=0.017), smartphone proficiency (A0R=4.703, 95% CI= 1.624—13.623, P=0.004) and prior smartphone usage to access medical information (AOR=5.472, 95% CI=2.790-10.732, P<0.001).

**Conclusion:**

Since smartphones were accessible to most primary care patients, and more than half of the patients were familiar with QR code scanning, QR code-based quality improvement projects can be used to improve services in our setting.

## Introduction

Quick response (QR) codes, initially developed by Denso Wave Incorporated, Japan, in 1994, are two-dimensional barcodes capable of storing extensive data.^1^ With a QR code reader on a camera-enabled mobile phone, physical QR codes can be seamlessly linked to digital information.^1^ In healthcare, QR codes can play pivotal roles in identifying patients, managing hospital assets and optimising various operations, including operating room protocols.^2,3^ They also facilitate streamlined data collection and analysis, enhancing care delivery efficiency and effectiveness for healthcare providers.^4^ Moreover, QR codes improve medical data accessibility and enhance the security of patient information exchange between healthcare systems.^5^

QR codes offer patients access to reliable health information, enhancing their understanding of medical conditions, treatments and procedures while fostering patient engagement.^6^ Additionally, they facilitate online appointment scheduling and contactless check-ins, reducing waiting times and enhancing overall patient satisfaction.^7^ Previous research has also demonstrated that the use of QR codes reduces self-administered medication errors.^8^ This comprehensive approach ultimately leads to improved health outcomes and lower healthcare costs. QR codes represent an innovative and versatile tool for patient care, empowering individuals to actively participate in their healthcare decisions and promoting informed choices.

The COVID-19 pandemic has significantly increased the utilisation of QR codes in various sectors, particularly within healthcare settings, as a means of minimising contact and reducing the risk of virus transmission. Given that COVID-19 spreads through respiratory droplets and contaminated surfaces, QR codes offer a timely solution for contactless information exchange, replacing traditional paper-based interactions. This transition towards contactless interactions has made QR codes a convenient and hygienic tool for accessing healthcare services and information. Consequently, numerous healthcare facilities have integrated QR codes for tasks such as patient vaccine registration, contact tracing, appointment scheduling and medical record access.^9^ Notably, QR codes have been instrumental in initiatives such as the United Kingdom (UK)’s National Health Service’s Test and Trace COVID-19 application, Malaysia’s MySejahtera application and China’s QR code system, facilitating symptom monitoring, contact tracing, isolation and quarantine, health status certification and travel authorisation during the pandemic.^9^

QR codes provide a range of advantages that contribute to their widespread adoption and utility. They facilitate seamless access to data through the simple act of scanning with camera-enabled mobile devices, thereby eliminating the need for physical contact and promoting hygiene in various contexts.^10^ Additionally, their production entails minimal expense, often being available at no cost, making them an economically viable solution for businesses and organisations of all sizes.^11^ The dynamic nature of QR codes also allows for effortless content updates without the requirement of generating new codes, enhancing their flexibility and adaptability.^1^ By replacing traditional paper-based methods, QR codes promote environmental sustainability by reducing paper consumption and waste.^10^ In sum, these attributes collectively render QR codes an accessible, practical, cost-effective and environmentally friendly tool for a wide array of applications.

Regional surveys regarding QR code utilisation within Malaysia are lacking. According to an online survey by Statista, the proportion of people using QR payments in Malaysia surged from 25% in 2021 to 61% in 2022.^12^ However, our investigation found no local surveys addressing QR code utilisation in the context of primary care. Overseas studies offer some insights; for instance, within the Australian general practice setting, 45% of patients required assistance with QR code usage as part of a study on a self-screening kiosk for atrial fibrillation.^13^ In contrast, a survey conducted in a UK orthodontics clinic revealed that 94% of patients found QR codes easy to use.^6^

In theory, the integration of QR codes within primary care settings presents numerous potential opportunities. However, its practical implementation may face challenges, particularly due to the diverse age demographics among primary care patients and variations in their technological proficiency. To date, there exists a dearth of research examining the prevalence of smartphone usage and familiarity with QR code scanning specifically among primary care patients in Malaysia. Thus, the primary objective of this study was to ascertain: (a) the level of ability of primary care patients to use a smartphone, their prior usage of a smartphone to obtain medical information and their familiarity with QR code scanning; (b) the factors influencing this familiarity; and, consequently, (c) the feasibility of instituting a QR code-based quality improvement initiative within our primary care facility.

## Methods

A cross-sectional survey was conducted among patients visiting a primary care centre at Universiti Malaya Medical Centre, Kuala Lumpur, from 9 January to 17 February 2023. This project was conducted as part of an audit process to enhance primary care services, particularly for health education purposes. Approval was obtained from the Research, Development and Innovation Department of Universiti Malaya Medical Centre (reference number: PPUM/RDI/400/09/001/34).

A sample size of 227 participants was determined using Kish’s formula, considering a desired confidence interval (CI) of 95%, an estimated proportion of patients familiar with QR code scanning of 82% and a margin of error of 5%.^6^ This calculation was based on data from a prior study conducted in a dental clinic. At that time, there were no studies about QR code usage in primary care, predating the commencement of our research. The prior study reported an 82% rate of familiarity with QR code usage among participants.^6^ Adjusting for a 20% non-response rate, we calculated a total required sample size of 272 participants.

**Figure 1:**
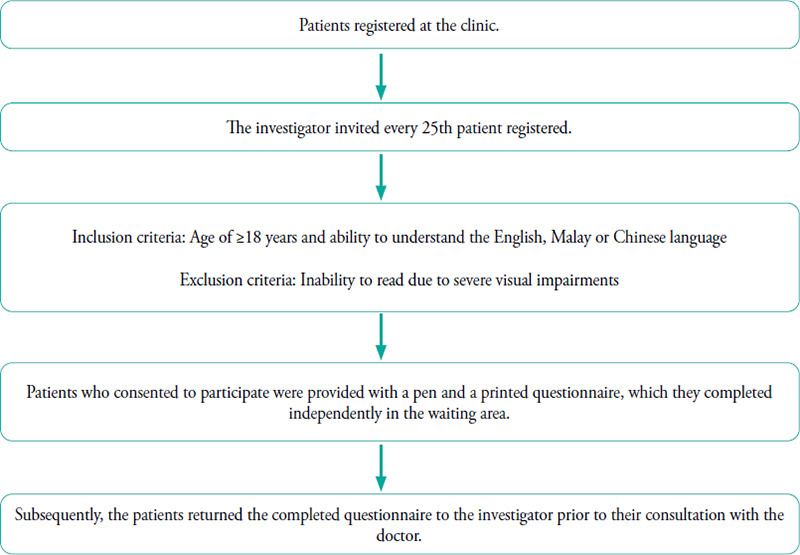
Flowchart of patient recruitment.

Figure 1 presents the flowchart of patient recruitment. Every 25th patient visiting the clinic from 8:00 AM to 1:00 PM who met the inclusion criteria was approached, allowing sufficient time for both the investigator and patients to complete the questionnaire, which took about 10 minutes.

**Figure 2:**
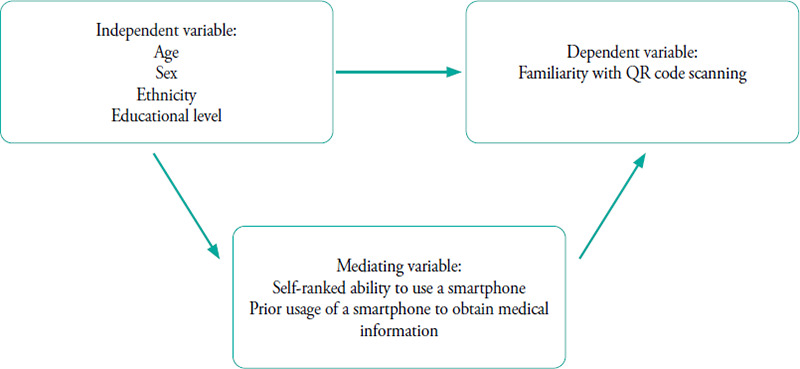
Conceptual framework.

Participants self-administered the printed questionnaire that assessed their sociodemographic background, type of mobile phone, perceived ability to use a smartphone, prior usage of a smartphone to access medical information and familiarity with utilising QR codes. The questionnaire was originally developed in English by a family medicine specialist, guided by a conceptual framework (Figure 2). Subsequently, a medical officer proficient in English, Chinese and Malay translated the questionnaire into Chinese and Malay versions. The questionnaire and its translations were reviewed by two other family medicine specialists who were fluent in native Chinese and Malay, respectively, and both proficient in English. Content validation was performed with input from three family medicine specialists within the department. Prior to the full implementation of the survey, the questionnaire underwent a pilot study in the same physical setting, involving 28 patients from 29 to 30 November 2022. Similar to the main study, the pilot study utilised printed questionnaires. There was no amendment made after the pilot study since all participants understood the questionnaire with no major comments.

For analysis, older persons were defined as individuals aged >65 years, following the 2019 United Nations World Population Ageing Report.^14^ The data were analysed using the Statistical Package for the Social Sciences version 26. The data distribution was skewed; thus, a non-parametric statistical analysis was conducted. The categorical data were reported as frequencies and percentages. The continuous data were described as medians and interquartile ranges (IQRs). The associations between the independent and dependent variables (familiarity with QR code scanning) were tested using logistic regression. Variables whose P-values were <0.25 in the univariate analysis were selected to fit a multiple logistic regression model, in which a P-value of <0.05 was considered significant.

## Results

**Figure 3:**
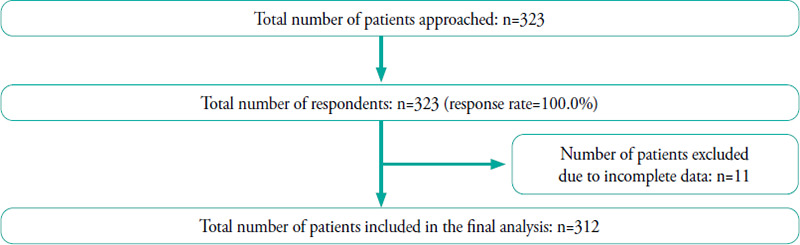
Flowchart of patient recruitment and exclusion.

Figure 3 illustrates the number of patients recruited and included in the analysis.

The median age of the participants was 57 years (IQR=4l—67), and 197 (63.1%) were women. In terms of ethnicity, 138 (44.2%) were Malays; 101 (32.4%), Chinese; and 68 (21.8%), Indians. Only 32 (10.2%) participants received primary education and below. The majority of the participants (n=261, 83.7%) self-ranked their proficiency in using a smartphone as average or good. More than half (n=181, 58.0%) had used a smartphone to obtain medical information, and 209 (67.0%) were familiar with QR code scanning. The independent, dependent and mediating variables among the 312 participants are summarised in Table 1.

**Table 1:** Independent, dependent and mediating variables among the participants (N=312).

Variable	n	%
Age, years		
Median (IQR)	57 (41-67)	
<65	206	66.0
≥65	106	34.0
Sex		
Male	115	36.9
Female	197	63.1
Ethnicity		
Malay	138	44.2
Chinese	101	32.4
Indian	68	21.8
Others	5	1.6
Educational level		
Primary or below	32	10.2
Secondary	131	42.0
Tertiary	149	47.8
Use of a smartphone		
Yes	282	90.4
No	30	9.6
Self-ranked ability to use a smartphone		
Poor	51	16.3
Average or good	261	83.7
Prior usage of a smartphone to obtain medical information		
Yes	181	58.0
No	131	42.0
Familiarity with QR code scanning		
Yes	209	67.0
No	103	33.0

Table 2 presents the factors associated with familiarity with QR code scanning. These factors included age of <65 years [adjusted odds ratio (AOR)=4.593, 95% CI=2.351-8.976, P<0.001], tertiary education (AOR=2.385, 95% CI=1.170—4.863, P=0.017), self-ranked average or good ability to use a smartphone (A0R=4.703, 95% CI= 1.624—13.623, P=0.004) and prior usage of a smartphone to obtain medical information (AOR=5.472, 95% CI=2.790-10.732, P<0.001).

**Table 2:** Univariate and multivariate analyses of the sociodemographic and other factors associated with familiarity with QR code scanning (N=312).

Variable	**Univariate**		P-value	**Multivariate**		P-value
	COR	95% CI		AOR	95% CI	
Age, year						
≥65 <65	Ref. 8.743	5.103-14.978	<0.001	4.593	2.351-8.976	<0.001
Sex						
Male Female	Ref. 0.600	0.361-0.995	0.048			
Ethnicity						
Others Malay Chinese Indian	Ref 1.820 1.314 0.844	0.292-11.326 0.209-8.240 0.132-5.382	0.521 0.771 0.858			
Educational level						
Secondary Primary or below Tertiary	Ref 0.396 5.621	0.174-0.902 3.138-10.067	0.027 <0.001	2.013 2.385	0.644-6.295 1.170-4.863	0.229 0.017
Smartphone proficiency						
Poor Average or good	Ref 18.006	8.027-40.390	<0.001	4.703	1.624-13.623	0.004
Prior usage of a smartphone to obtain medical information						
No Yes	Ref 11.708	6.632-20.669	<0.001	5.472	2.790-10.732	<0.001

Variables demonstrating a P-value of <0.05 in the multivariate analysis are shown in bold. COR, crude odds ratio; AOR, adjusted odds ratio; CI, confidence interval

## Discussion

The global use of QR codes during the COVID-19 pandemic for contact tracing has established a lasting legacy, resulting in widespread familiarity with QR code scanning among the majority of people.^9^ Prior to our study, no research has been conducted on the utilisation of QR codes within primary care settings in Malaysia. This study represents the inaugural investigation into this area of inquiry.

In the present study, the majority (44.2%) of our patients were Malays, followed by Chinese (32.4%) and Indians (21.8%), reflecting the country’s demographic. The rate of familiarity with QR code scanning among our patients stood at 67.0%. Our results mirror those of a recent study conducted in an Australian general practice setting, wherein 55% of patients did not require assistance with QR code utilisation.^13^ This finding indicates a potential feasibility for implementing QR code-based projects within our clinic.

Other factors impacting the feasibility of a QR code-based project include its ease of integration with existing clinic management systems, its cost of generation and maintenance, the level of staff training and technical support required and the availability of the internet. QR codes are inherently easy to use, flexible, affordable and adaptable, making them highly feasible tools for use in various projects.^1^ The widespread adoption of QR codes during the COVID-19 pandemic, including for restaurant menus, has reduced the need for extensive staff training and technical support.^10^ In Malaysia, the proportion of mobile internet users doubled from 48.62% in 2014 to 88.86% in 2023.^15^ By early 2024, Malaysia had 44.55 million active cellular mobile connections, representing 129.2% of the total population.^16^ These factors collectively enhance the feasibility of QR code-based projects.

The feasibility of a QR code-based project can also be influenced by patient preferences and accessibility for specific groups, such as individuals with visual impairments. While a 2016 survey in Sweden showed a preference for printed medication leaflets, video or interactive web content deliverable via QR codes may be more favoured.^17^ Studies in Australia have found that portable video media for surgical information and digital video discs for fall prevention improve patient satisfaction and engagement over traditional methods.^18,19^ For individuals with visual impairments, QR codes with text-to-speech technology enhance accessibility by converting text to spoken words.

Our study identified high-risk groups less familiar with QR code scanning, including individuals aged ≥65 years, those who selfreported poor smartphone usage ability and those with no prior experience using smartphones for medical information. Older adults may struggle with smartphone applications due to sensory limitations (small fonts/screens), cognitive challenges (complex interfaces), motor skill issues, financial constraints and a lack of technological familiarity.^20^ Conversely, patients with prior experience using smartphones for medical information are more familiar with QR codes, given their increasing use in healthcare.^6^ Nevertheless, advancements in smartphone design, such as customisable displays, larger screens, voice commands and simplified interfaces, are increasingly overcoming these barriers.^21^ Studies have shown growing smartphone adoption and satisfaction among older adults. In Iran, older individuals reported acceptable usability and satisfaction scores with smartphones in 2019.^22^ In the UK, 58.5% of older individuals owned a smartphone in 2020, while 16.2% intended to acquire one.^23^ In Malaysia, 82.3% of older adults were smartphone users in 2021.^24^ A study conducted in 2016 found that a longer duration of smartphone use (>2 years vs 6 months to 2 years vs <6 months) improved the ability of older adults to use the internet (P<0.001) and access content (P=0.001).^25^ In another study, older individuals in the United States were found to prefer scanning QR codes over typing URLs to avoid typing errors.^26^ Despite age barriers, most older individuals own smartphones, demonstrate the ability to use them and have the capacity to improve their skills over time.

Our study found that the individuals without tertiary education were less familiar with QR code scanning. While there is limited literature directly linking the educational level with the ability to scan QR codes, previous reports are inconsistent regarding the association between educational attainment and the use of mobile devices for health purposes.^25,27^ Nevertheless, an increasing number of Malaysians are expected to receive tertiary education as a result of policies such as the Malaysian Education Blueprint 2013-2025 and The National Higher Education Strategic Plan Beyond 2020.^28^ Malaysia has more than 942,200 students enrolled in 595 universities and colleges, with 60% of tertiary education funded by the government in 2014.^28^ The future Malaysian population is expected to become increasingly familiar with QR code scanning. This trend suggests that QR code-based quality improvement projects represent a sustainable direction that will benefit future generations over the long term.

We recommend implementing QR code-based quality improvement projects to further enhance patients’ ability to use smartphones because using these devices positively affects the ability of older individuals to use the internet (P<0.01), participate in community activities (P<0.01) and engage in communication (P<0.01).^25^ Systematic reviews have shown that using mobile devices for healthcare delivery (i.e. mHealth interventions) enhances treatment adherence in 56%-65% of studies involving patients with chronic diseases and improves disease-specific clinical outcomes in 39% of studies encompassing conditions such as diabetes mellitus, cardiovascular disease and chronic lung disease.^29^ Mobile-device engagement programmes, education and eHealth services may be beneficial to overcome the barriers faced in the implementation of QR code-based quality improvement projects.

Subsequent to our research, our clinic has initiated QR code-based quality improvement projects. Among these initiatives is the Project Informasi Laluan & Transit, where patients utilise QR codes to access maps guiding them to various hospital locations from our clinic, enhancing patient navigation within the facility. Additionally, we have introduced the Scan Me Project, allowing patients to access medical educational materials by scanning QR codes on posters within our clinic. This approach provides convenient access to valuable health information, enhancing patient understanding of medical topics and promoting education within our healthcare environment.

Our study has notable limitations. Initially conceived as an audit to assess the feasibility of QR code-based quality improvement projects in our clinic, the study lacked the depth of formal research. We missed incorporating variables such as income, underlying medical conditions, occupation and patient preferences in our questionnaire. Additionally, incomplete questionnaire responses could have been minimised with investigator oversight. The generalisability of our findings may also be limited, as our local population is more affluent and predominantly working class compared to non-urban populations. Nevertheless, a low income may not significantly deter smartphone and QR code usage. According to a 2021 survey, 91.6% of Malaysians earning RM 1000 or less per month owned smartphones, indicating the increased affordability of these devices in Malaysia.^24^ We also inferred familiarity with QR code scanning as the ability to scan QR codes without assistance. Although they are not directly the same, we believe that patients’ self-reported familiarity with QR code scanning reflects their ability to do so independently because the term ‘familiarity’ encompasses nuances associated with better recognition accuracy, source accuracy, confidence and response time compared to the term ‘knowing’.^30^

Additional research is warranted to assess the effectiveness of QR code-based quality improvement projects. This includes comparing QR code-based patient education with traditional leaflet-based education regarding patient understanding, information retention and patient preference. Furthermore, there is a scarcity of studies investigating whether the contactless nature of QR code-based projects contributes to reducing the transmission rates of infectious diseases and its potential impact on public health outcomes.

In summary, our study shows that most Malaysians have smartphones, enabling QR code scanning and access to medical information online. However, older adults and those without tertiary education may face barriers due to lesser technology adoption. Mobile-device engagement programmes, education and eHealth services may be beneficial in easing the transition to QR code based quality improvement projects. This approach ensures that patients are prepared for the evolving technological landscape of healthcare.

## Data sharing statement

The data utilised in this study are not open for external access or sharing. For further inquiries regarding the data, please contact Dr Yeoh Zi Yi at yeohziyi@ummc.edu.my.

**How does this paper make a difference in general practice?**

- Our study is the first to explore quick response (QR) code utilisation in Malaysian primary care.
- Most patients use smartphones and are familiar with QR codes, suggesting an opportunity to enhance health education and communication in primary care.
- Older patients and patients without tertiary education may be less familiar with QR code scanning; mobile-device engagement programmes, education and eHealth services may be beneficial.
- Potential barriers to QR code adoption are identified, such as age and education, urging further investigation into strategies for equitable access to digital health resources.
- Following our study, our clinic has launched QR code-based quality improvement projects.
